# Integration of leaf traits supports the current circumscription of *Afgekia* Craib. and *Padbruggea* Miq. (Fabaceae, Wisterieae)

**DOI:** 10.3897/phytokeys.270.181424

**Published:** 2026-02-05

**Authors:** Punvarit Boonprajan, Yotsawate Sirichamorn

**Affiliations:** 1 Department of Biology, Faculty of Science, Silpakorn University, Sanam Chandra Palace Campus, Nakhon Pathom 73000, Thailand Silpakorn University Nakhon Pathom Thailand

**Keywords:** *
Afgekia
*, leaf anatomy, leaf venation, *
Padbruggea
*, taxonomic significance, trichomes

## Abstract

Accurate delimitation of *Afgekia* and *Padbruggea*, two morphologically similar genera in the tribe Wisterieae (Fabaceae), has long posed challenges by overlapping vegetative traits, particularly in the case of *P. filipes*, which had been historically misclassified under *Afgekia*. This study aims to clarify their generic boundaries by integrating comparative analyses of leaf traits, including venation architecture, trichome morphology, epidermal and stomatal characteristics, and transverse anatomy. Microscopic and image-based assessments were conducted across populations of *A. mahidoliae*, *A. sericea*, *P. dasyphylla*, and *P. filipes*. Results revealed stable inter- and infrageneric differences in leaf venation patterns, accessory vascular bundle types, trichome diversity, stomatal distribution, and presence of idioblasts and calcium oxalate crystals. Notably, *P. filipes* displayed diagnostic anatomical features that aligned more closely with *Padbruggea*, supporting its recent transfer from *Afgekia* based on phylogenetic studies. Despite some environmental inconsistencies, the observed traits showed high species-level stability across populations, underscoring their taxonomic significance. This integrative anatomical approach lends support to the current circumscription of both genera and suggests the continued relevance of vegetative anatomy as a complementary line of evidence to molecular data, potentially aiding the refinement of taxonomic concepts within Fabaceae.

## Introduction

Taxonomic clarity of biological entities requires rigorous investigation, critical evaluation, and confirmation from multiple lines of evidence. Scientific and technological advances have played a pivotal role in facilitating taxonomic revisions. Traditionally, classifications were based primarily on morphological characters. However, the rise of molecular phylogenetics and a monophyletic framework has shifted the focus toward integrating genetic data to improve taxonomic resolution. This shift has helped resolve inconsistencies arising from homoplastic traits and morphological similarities that evolved independently rather than from a shared ancestry (Went 1971; Yang et al. 2015; Naramoto et al. 2019; Wessinger and Hileman 2020), as seen in the genus *Callerya* Endl. (Fabaceae) and its allied genera, which have undergone substantial taxonomic reassessment and are subjects of ongoing investigation.

Over the past two decades, taxonomists have revisited the generic limits within the *Callerya* group, leading to ongoing debates and reclassifications (Endlicher 1843; Miquel 1855; Dunn 1911, 1912; Craib 1928; Geesink 1984; Schot 1994; Hu et al. 2000, 2002; Hu and Chang 2003; Jansen et al. 2008; Li et al. 2014; Compton et al. 2019; Duan et al. 2021a, b). Among these, Compton et al. (2019) provides the most comprehensive and widely adopted framework, integrating detailed morphological evidence with molecular phylogenetic analyses. That study reinstated or elevated several taxa previously treated under *Callerya* to the generic level, resulting in a more phylogenetically coherent circumscription of genera within the *Callerya* complex. This integrative approach has clarified long-standing ambiguities, particularly between *Afgekia* Craib and *Padbruggea* Miq., which have historically been difficult to delimit. (The broader phylogenetic framework and current species circumscription of each genus are summarized in Suppl. material 1: table SS1 and Suppl. material 2)

Modern phylogenetic studies have demonstrated that *Padbruggea* forms a distinct clade, closely related to *Austrocallerya* J.Compton & Schrire and *Wisteria* Nutt., and clearly separated from *Afgekia*. In particular, the study by Compton et al. (2019) consistently recovered *Afgekia* within a broader clade comprising *Callerya*, *Kanburia* J.Compton, Mattapha, Sirich. & Schrire, *Serawaia* J.Compton & Schrire, and *Whitfordiodendron* Elmer (Compton et al. 2019; Duan et al. 2021a, b). Although genera *Afgekia* and *Padbruggea* members share a historical and taxonomic relationship, with species exchange being reassigned between them over time (Craib 1928; Geesink 1984; Lôc and Vidal 2001; Jansen et al. 2008; Wei and Pedley 2010), recent evidence, along with observations by several taxonomists such as Geesink (1984) and Sirichamorn (2006), has helped to clarify the generic boundaries between the two genera. This separation is supported by morphological distinctions, such as from the inflorescence architecture, number and structure of floral callosities, and pod morphology, and molecular data derived from chloroplast and nuclear markers. Notably, *Afgekia filipes* (Dunn) R.Geesink has been reassigned to *Padbruggea* based on its closer affinity to the morphological and genetic traits characteristic of that genus (Wei and Pedley 2010; Compton et al. 2019; Duan et al. 2021a, b). *Afgekia* is now recognized as comprising two species (*A. mahidoliae* B.L.Burtt & Chermsir. and *A. sericea* Craib), whereas *Padbruggea* includes three species (*P. dasyphylla* Miq., *P. maingayi* (Baker) Dunn, and *P. filipes*) (Compton et al. 2019).

While molecular and morphological data provide a robust framework for taxonomic placement, the practical identification of *Afgekia* and *Padbruggea* remains problematic, especially in the absence of reproductive structures. This is a frequent issue in herbarium specimens and field surveys, where flowers and fruits are often missing. Moreover, intrageneric identification is also challenging, as closely related species within each genus often exhibit only subtle morphological differences that may be difficult to discern. Lack of field-based observations is also problematic for genus-level identification. For instance, older stems of *Padbruggea* often exude a reddish latex when cut, a feature not commonly observed in *Afgekia*. Although both genera occur in Thailand, they tend to occupy different ecological niches: *Padbruggea* typically inhabits more humid forest environments, while *Afgekia* is more frequently found in drier, open areas, including limestone outcrops. In “Flore du Cambodge, du Laos et du Viêtnam”, Lôc and Vidal (2001) reported the distribution of *A. sericea* in Thailand and Laos, and *A. mahidoliae* in Thailand, Laos, and Vietnam. However, current evidence suggests that the genus *Afgekia* may, in fact, be endemic to Thailand. A known population of *A. sericea* occurs near the Thai–Cambodian border, in the vicinity of Prasat Hin Phanom Rung Historical Park, Buriram Province. Therefore, its presence in Cambodia remains plausible, although it has yet to be formally documented. Moreover, some herbarium specimens from Laos and Vietnam housed at the Muséum national d’Histoire naturelle (P) in Paris have been misidentified as *Afgekia*, when in fact they are referable to *Padbruggea* spp. These specimens were likely cited in the flora in error (Sirichamorn, pers. obs.). In such cases, additional micro-morphological and anatomical traits, particularly from vegetative parts, can serve as critical tools for taxonomic decision-making (Metcalfe and Chalk 1950, 1979; Yang and Lin 2005; Zou et al. 2008; Andres-Hernandez and Terrazas 2009; Silva et al. 2018; Song et al. 2020; Silva et al. 2022; Zhao et al. 2022b).

Leaves, as primary vegetative organs exposed to the aerial environment, reflect long-term adaptive strategies and exhibit consistent morphological features (Niinemets et al. 2009; Scafaro et al. 2011; Terashima et al. 2011; Wang et al. 2021). Leaf traits have long been recognized as essential tools in plant taxonomy, aiding both infrageneric classification and species delimitation. Surface features, transverse anatomical sections, and architecture of venation have proven particularly useful in distinguishing taxa when unavailable reproductive data (Uphof 1962; Stenglein et al. 2003; Yang and Lin 2005; Cardoso and Sajo 2006; Ellis et al. 2009; Chukwuma et al. 2014; Devecchi et al. 2014; Silva et al. 2018; Thacker et al. 2021; Rajput et al. 2021). These characteristics often provide valuable insights into phylogenetic relationships, especially within complex families like Fabaceae (Chukwuma et al. 2014; Devecchi et al. 2014; Silva et al. 2018; Silva et al. 2022; Zhao et al. 2022b).

Despite their importance, micro-observation of leaf structures is limited by practical constraints. Leaf is typically preserved under dry conditions, making it nearly impossible to obtain data from herbarium specimens. Consequently, most taxonomic literature is either superficial or neglected. For example, reports frequently mention either hairy or glabrous of a specific characteristic or describe their texture with modifiers like sericeous, strigose, hirsute, or velvety (Mattapha and Sirichamorn 2020). Given this gap, comprehensive anatomical studies of *Afgekia* and *Padbruggea* leaves are still lacking. Therefore, this study aims to conduct a comprehensive comparative investigation of leaf anatomical characters in *Afgekia* and *Padbruggea*, emphasizing both inter- and intra-generic variation. Through detailed analyses of leaf epidermal, micro-morphological, and transverse anatomical traits, we assess their taxonomic significance and identify reliable vegetative characters that facilitate generic delimitation and species identification, particularly when reproductive structures are absent.

## Material and methods

### Specimen collection and morphological identification:

Specimens of *Afgekia* and *Padbruggea* were collected from various sites and habitats across Thailand, as summarized in Table 1. Voucher specimens were prepared following standard taxonomic protocols and deposited at the Herbarium of Mahidol University (PBM). For micro-morphological and anatomical investigation, mature leaves were collected from three individuals per population for each species. Samples were preserved in 70% ethanol for subsequent histological analysis.

**Table 1.** Information on plant materials examined in this study.

Accession	Species	Locality-Province	Voucher (Herbarium)
*Afgekia* Craib			
1-AS	*A. sericea* Craib	Nakhon Ratchasima	YSM2024-18 (PBM)
2-AS		Buri Ram	YSM2024-19 (PBM)
3-AS		Nakhon Pathom	P. Boonprajan-19 (PBM)
4-AM	*A. mahidoliae*	Kanchanaburi	YSM2024-11 (PBM)
5-AM	B.L.Burtt & Chermsir.	Nakhon Pathom	P. Boonprajan-15 (PBM)
*Padbruggea* Miq.			
6-PD	*P. dasyphylla* Miq.	Nakhon Si Thammarat	YSM2021-29 (PBM)
7-PD		Songkhla	YSM2019-9 (PBM)
8-PD		Phangnga	YSM2021-20 (PBM)
9-PF	*P. filipes* Craib	Kanchanaburi	YSM2021-25 (PBM)
10-PF		Chiang Mai	YSM2019-14 (PBM)

Species identification was based on detailed morphological assessments, compared with descriptions in the Flora of Thailand (Mattapha and Sirichamorn 2020) and high-resolution images of specimens and type specimens from major online repositories (e.g., K, L, P).

### Leaflet epidermal preparation:

Epidermal peels were manually prepared by gently scraping the opposite surface of the leaflet with a razor blade and fine brush to expose the targeted epidermis. The samples were bleached in commercial sodium hypochlorite until translucent, rinsed thoroughly with distilled water, and preserved in 70% ethanol. Each individual was represented by at least three replicate peels collected from different leaves. Permanent slides were prepared by staining with 0.1% Fast Green, followed by dehydration through a graded ethanol series. Samples were then cleared in a 1:1 solution of ethanol and xylene, immersed in pure xylene, and mounted using DPX mounting medium.

### Transverse sectioning of leaf structures:

Leaf segments from the petiole of the pulvinus and petiolule, petiole, rachis, midrib, leaflet blade, and leaflet margin were dehydrated through a graded ethanol series and embedded in paraplast following tertiary butyl alcohol (TBA) infiltration. Tissues were sectioned at 10–18 μm thickness using a rotary microtome (Leica RM2145). Ribbon sections were floated on warm water, affixed to microscope slides, stained with 1% Safranin and 0.1% Fast Green, dehydrated, cleared in xylene, and mounted with DPX.

### Micro-morphology of trichomes and leaf architecture:

Paradermal sections were prepared from the same leaf segments used for transverse sectioning, excluding the midrib region, for trichome examination. In addition, terminal and lateral leaflets of compound leaves, prepared for leaf architecture examination, were manually brushed to remove trichomes. All specimens were cleared and stained with 0.1% Fast Green. Excess stain was removed by gently rinsing with 70% ethanol. The processed samples were subsequently preserved in glycerol and examined under light microscopy for detailed morphological observation.

All leaflet epidermal surfaces and transverse sections were examined and digitally photographed using an Olympus BX53 light microscope fitted with an Olympus DP27 camera. Trichome morphology and leaf architecture were studied under a Nikon SMZ800N stereomicroscope equipped with a Nikon DS-Fi3 camera. Anatomical and micro-morphological terminology follows the conventions outlined by Dilcher (1974), Metcalfe and Chalk (1979), and Ellis et al. (2009). Descriptive anatomical features were recorded for each species and compared qualitatively across taxa to assess interspecific and intergeneric variation, based on the presence or absence, structural patterns, and relative development of epidermal, trichome, and transverse section traits. Quantitative anatomical characters were measured from digital images using ImageJ software (version 1.53t; NIH, USA), with at least five replicates per character per species. Measurements were expressed as means ± standard deviations. Quantitative data were used to support and corroborate qualitative comparisons among taxa. Raw measurement data were processed and tabulated using Microsoft Excel 2021. No inferential statistical analyses were performed, as the study aimed to document and compare diagnostic anatomical characters rather than to test statistical differences among taxa.

## Results

**Figure 1.**
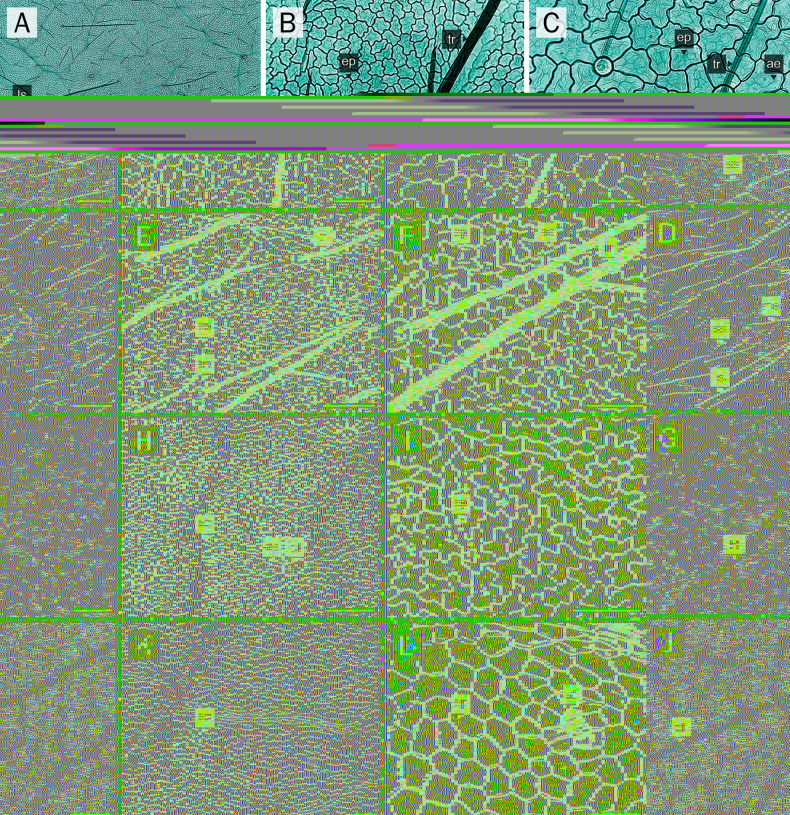
Comparative anatomical characters of adaxial leaflet epidermis. **A–C**. *A. mahidoliae*; **D–F**. *A. sericea*; **G–I**. *P. dasyphylla*; **J–L**. *P. filipes*. Abbreviations: ae = adjacent ep of basal cell; bt = basal cell of tr; ep = epidermal cell; ls = large straight; ms = medium straight; ss = small straight; st = stomata; tr = eglandular trichome. Scale bars: 100 µm (**B, E, H, K**); 50 µm (**C, F, I, L**); 500 µm (**A, D, G, J**).

**Figure 2.**
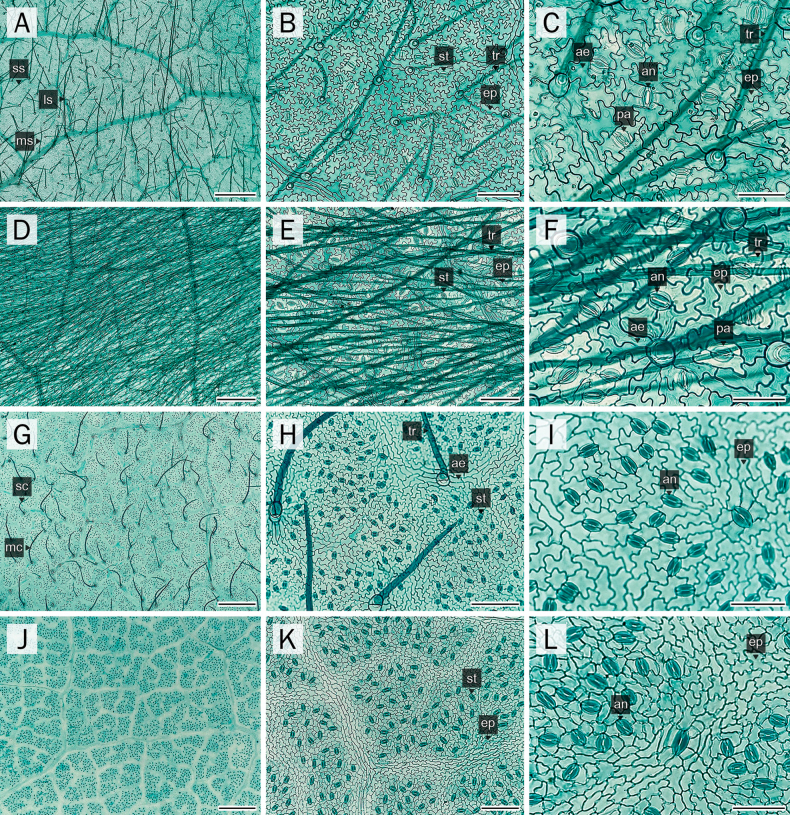
Comparative anatomical characters of abaxial leaflet epidermis. **A–C**. *A. mahidoliae*; **D–F**. *A. sericea*; **G–I**. *P. dasyphylla*; **J–L**. *P. filipes*. Abbreviations: ae = adjacent ep of basal cell; an = anomocytic stomata; bt = basal cell of tr; ep = epidermal cell; ls = large straight; ms = medium straight; pa = paracytic stomata; sc = small curly; ss = small straight; tr = eglandular trichome. Scale bars: 50 µm (**C, F, I, L**); 100 µm (**B, E, H, K**); 500 µm (**A, D, G, J**).

**Figure 3.**
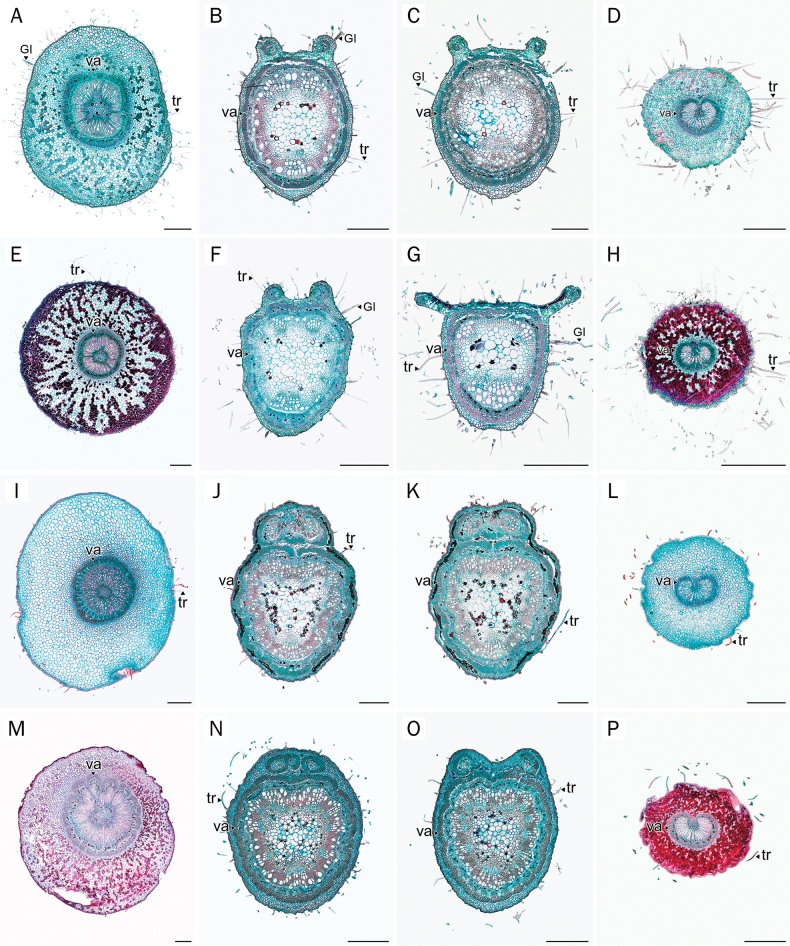
Comparative anatomical characters of leaf transverse sections. **A, E, I, M**. Petiole-pulvinus; **B, F, J, N**. Petiole; **C, G, K, O**. Rachis; **D, H, L, P**. Petiolule-pulvinus. **A–D**. *A. mahidoliae*; **E–H**. *A. sericea*; **I–L**. *P. dasyphylla*; **M–P**. *P. filipes*. Abbreviations: GI = glandular trichome type I; tr = eglandular trichome; va = vascular tissue. Scale bars: 500 µm (**A–P**).

**Figure 4.**
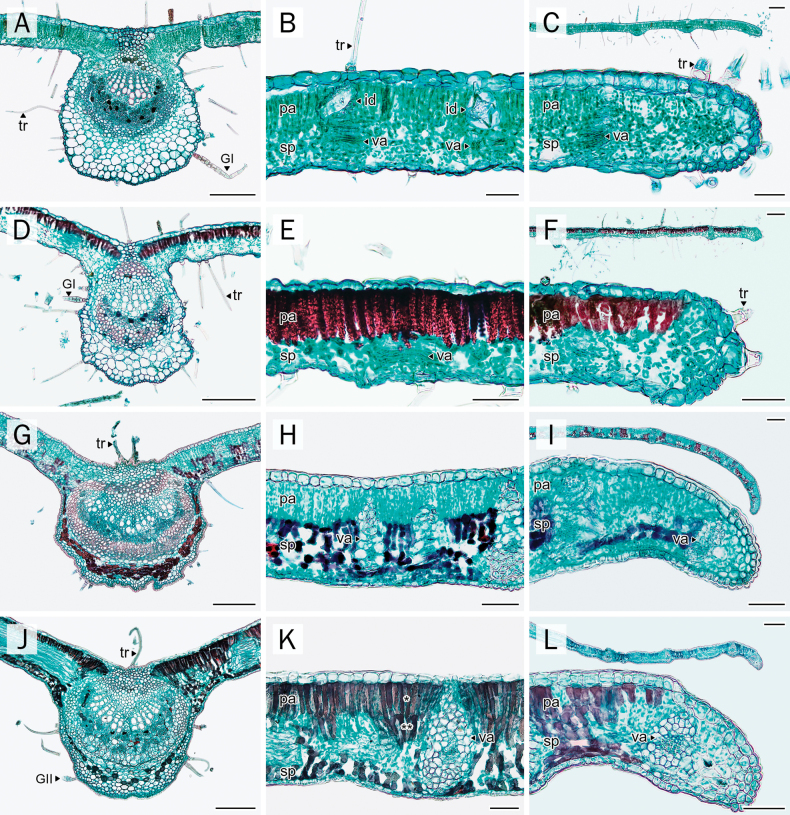
Comparative anatomical characters of leaflet transverse sections. **A, D, G, J**. Midrib; **B, E, H, K**. Leaflet blade; **C, F, I, L**. Leaflet margin. **A–C**. *A. mahidoliae*; **D–F**. *A. sericea*; **G–I**. *P. dasyphylla*; **J–L**. *P. filipes*. Abbreviations: pa = palisade parenchyma layer; GI = glandular trichome type I; GII = glandular trichome type II; id = idioblast cell with crystal prism; sp = spongy parenchyma layer; tr = eglandular trichome; va = vascular tissue; (*) = palisade parenchyma layer 1; (**) = palisade parenchyma layer 2. Scale bars: 50 µm (**B, E, H, K** (**C, F, I, L**; below)); 200 µm (**A, D, G, J** (**C, F, I, L**; above)).

**Figure 5.**
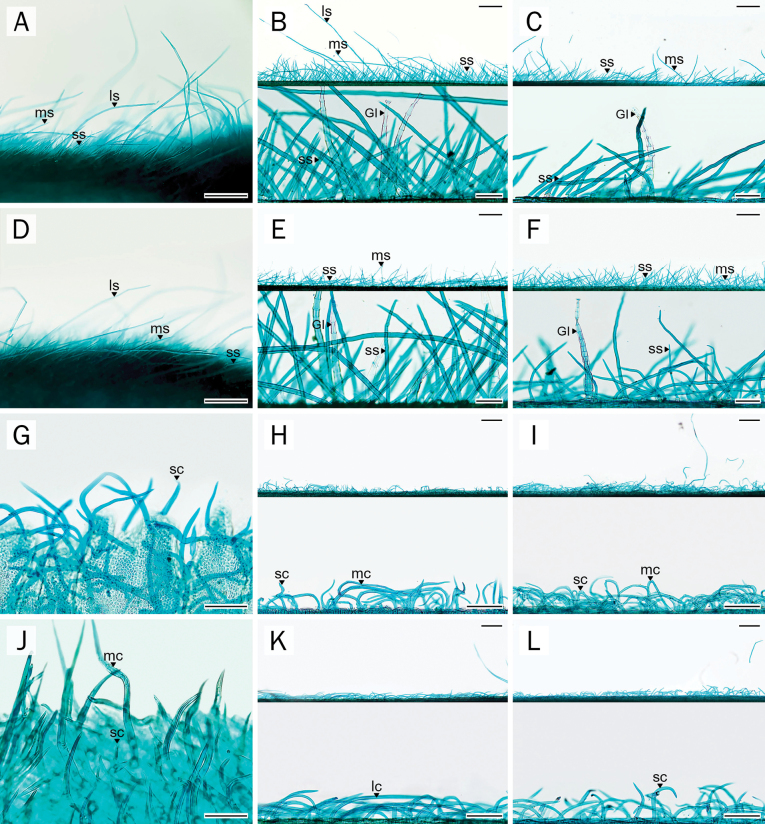
Comparative micro-morphology characters of trichomes. **A, D, G, J**. Petiole-pulvinus; **B, E, H, K**. Petiole; **C, F, I, L**. Rachis; **A–C**. *A. mahidoliae*; **D–F**. *A. sericea*; **G–I**. *P. dasyphylla*; **J–L**. *P. filipes*. Abbreviations: GI = glandular trichomes type I; lc = large curly; ls = large straight; mc = medium curly; ms = medium straight; sc = small curly; ss = small straight. Scale bars: 100 µm (**B, C, E, F, I, L**; below); 200 µm (**A, D, G, J** (**H, K**; below)); 500 µm (**B, C, E, F, H, I, K, L**; above).

**Figure 6.**
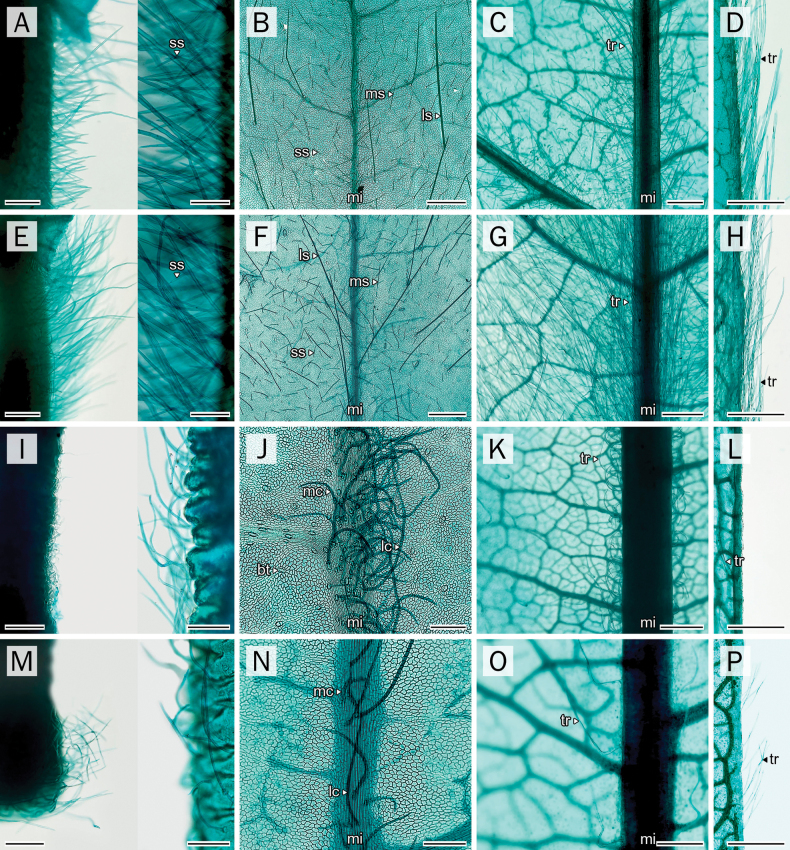
Comparative micro-morphology characters of trichomes. **A, E, I, M**. Petiolule-pulvinus; **B, F, J, N**. Midrib-adaxial; **C, G, K, O**. Midrib-abaxial; **D, H, L, P**. Leaflet margin. **A–D**. *A. mahidoliae*; **E–H**. *A. sericea*; **I–L**. *P. dasyphylla*; **M–P**. *P. filipes*. Abbreviations: lc = large curly; ls = large straight; mc = medium curly; mi = midrib; ms = medium straight; sc = small curly; ss = small straight. Scale bars: 200 µm ((**A, E, L, M**; right), **D, H, J, L, N, P**); 500 µm ((**A, E, L, M**; left), **B, C, F, G, K, O**).

**Figure 7.**
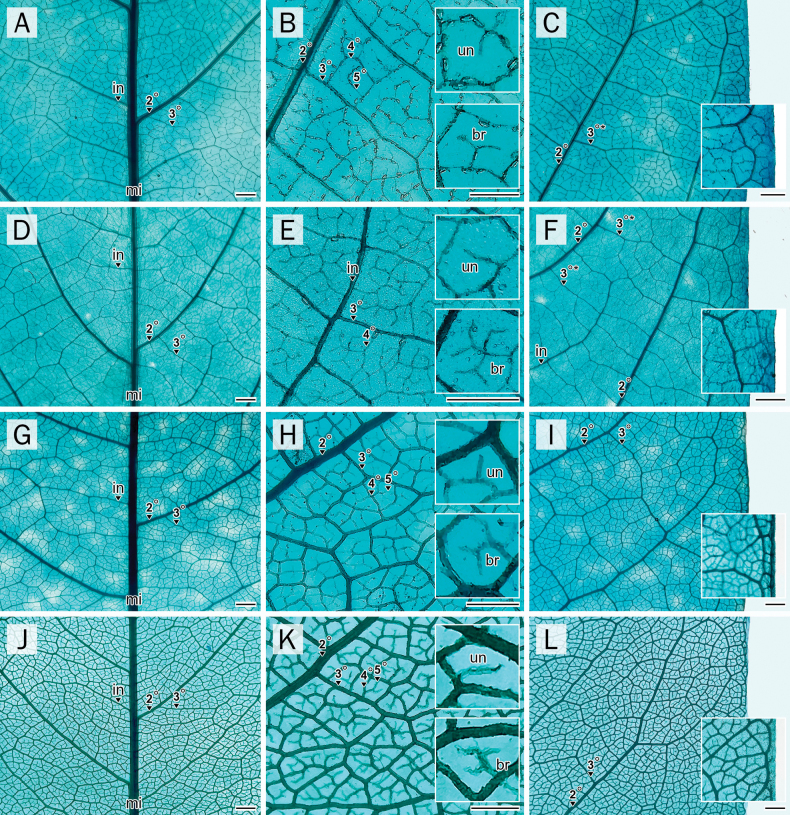
Comparative characters of leaf architecture. **A, D, G, J**. Midrib; **B, E, H, K**. Lamina; **C, F, I, L**. Margin; **A–C**. *A. mahidoliae*; **D–F**. *A. sericea*; **G–I**. *P. dasyphylla*; **J–L**. *P. filipes*. Abbreviations: br = FEVs branched; in = intersecondary veins; mi = midrib; un = FEVs unbranched; 2° = secondary vein; 3° = tertiary vein; 3°^*^ = tertiary vein percurrent; 4° = quaternary vein; 5° = quinternary vein. Scale bar: 1 mm (**A–L**).

**Figure 8.**
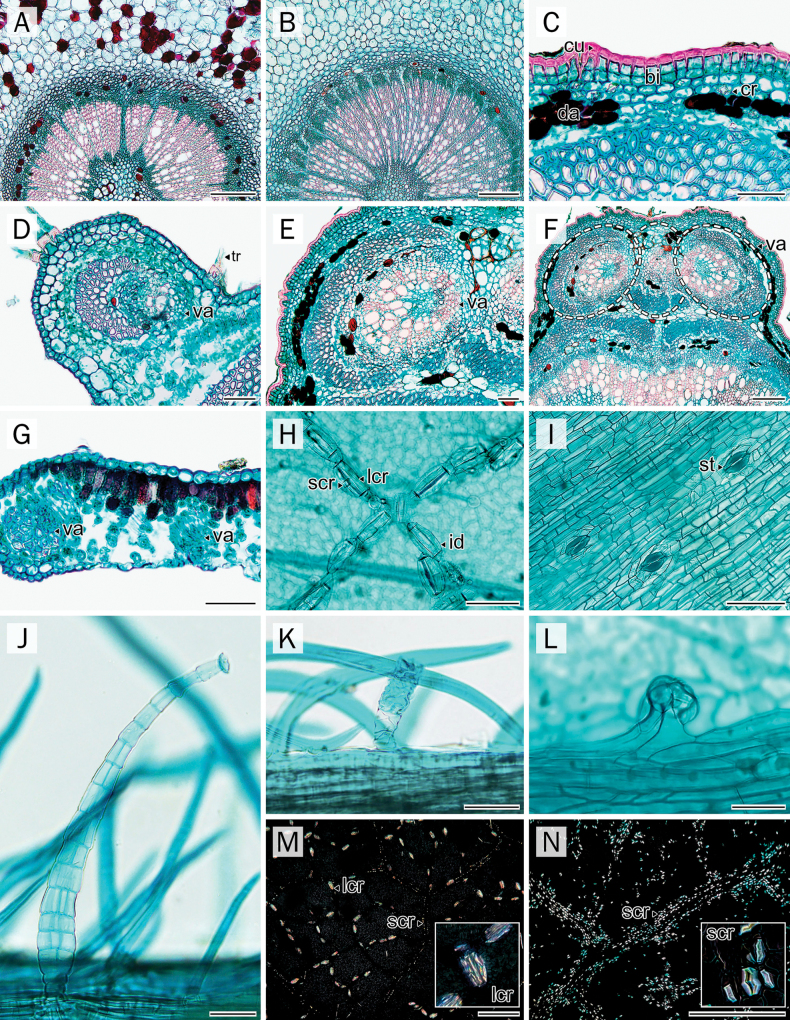
Compound (**A–I**), stereo (**J–L**), and polarized (**M, N**) light microscope image of micromorphology and anatomy characteristics. **A, B**. Petiole-pulvinus; **C**. Petiole; **D–G**. Ridge of rachis; **H**. Leaflet lamina; **I**. Midrib; **J**. Glandular trichomes type I; **K**. Glandular trichomes type II; **L**. Glandular trichomes type III; **M, N**. Distribution of CaOx on leaflet; **A, G, L**. *A. sericea*; **B, C, E, F**. *P. dasyphylla*; **D, H, J, M**. *A. mahidoliae*; **I, K, N**. *P. filipes*. Abbreviations: cu = cuticle layer; da = dark-deposited; id = idioblast cell with crystal prism; lcr = large crystal prism; scr = small crystal prism; tr = eglandular trichome; va = vascular tissue; (*) = epidermal cell layer 1; (**) = epidermal cell layer 2. Scale bars: 50 µm (**C–G, H–L**); 200 µm (**A, B, F, I, K, L, N**); 500 µm (**M**).

Anatomical, micro-morphological, and leaf architectural investigations revealed a high degree of consistency among individuals of the same species across different populations. Diagnostic traits were observed across epidermal features, internal leaf structure, trichome types and distributions, and venation patterns, supporting taxonomic distinctions at both the genus and species levels (Tables 2–4; Figs 1, 2, 3, 4, 5, 6, 7, 8; Suppl. material 1: tables S2–S4).

### Epidermal cell wall

The anticlinal cell walls ranged from slightly polygonal to sinuate (Figs 1, 2). On the adaxial surface, samples of *P. filipes* generally exhibited polygonal to irregular epidermal cells with straight anticlinal walls (Fig. 1J–L), whereas *P. dasyphylla* and both *Afgekia* species (Fig. 1A–I) showed undulate, polygonal to jigsaw puzzle-shaped walls. On the abaxial surface, all samples from both genera exhibited epidermal cells with undulate anticlinal walls (Fig. 2). Epidermal cells on the adaxial surface were also generally larger than those on the abaxial surface (Table 2).

**Table 2.** Comparison of selected leaf epidermal characters (see Suppl. material 1: table SS2 for additional characters).

Characters	Species			
	*A. mahidoliae*	*A. sericea*	*P. dasyphylla*	*P. filipes*
**Adaxial surface** (Fig. 1)				
Patterns of epidermal cell walls	Undulate	Undulate	Undulate	Straight
Area of epidermal cell (µm^2^)	1351.35±347.94	1097.54±184.82	562.30±169.28	669.77±228.02
Stomatal presence (type)	Absent	Absent	Absent	Present (rarely, AN)
**Abaxial surface** (Fig. 2)				
Patterns of epidermal cell walls	Undulate	Undulate	Undulate	Undulate
Area of epidermal cell (µm^2^)	1160.54±155.01	862.56±113.13	368.72±124.02	251.01±74.36
Stomatal type	PA and AN (rarely)	PA and AN (rarely)	AN	AN
Stomatal presence on midrib and minor veins	Absent	Absent	Absent	Present
Stomatal density (per mm^2^)	129.33±10.07	169.33±11.02	286.33±25.58	294.67±18.48
Stomatal index (SI)	15.48±0.88	13.86±0.73	14.55±1.71	13.30±0.74

Note: An, anomocytic stomata; Pa, paracytic stomata

Stomata were restricted to the abaxial surface (hypostomatic) in *A. mahidoliae*, *A. sericea*, and *P. dasyphylla* (Fig. 2C, F, I). Conversely, they were also rarely observed on the adaxial surface (amphistomatic) in *P. filipes*, and notably, stomatal distribution extended across the midrib region (Fig. 8I). Two stomatal types, anomocytic and paracytic, were observed in *Afgekia* (Fig. 2C, F), whereas only the anomocytic type was present in *Padbruggea* (Fig. 2I, L). Stomatal density was notably higher in *Padbruggea* (142–316 stomata per mm^2^) than in *Afgekia* (120–182 stomata per mm^2^). Meanwhile, the highest stomatal index was recorded in *A. mahidoliae*, and the lowest in *P. filipes* (Table 2).

### Petiole-pulvinus

The outlines of the petiole-pulvinus were generally rounded, particularly in *A. sericea* and *P. filipes*, while *A. mahidoliae* and *P. dasyphylla* tended to exhibit more elliptical shapes, with the x-axis being slightly narrower than the y-axis (Table 3; Fig. 3).

**Table 3.** Comparison of selected leaf transverse section characters (see Suppl. material 1: table SS3 for additional characters).

Characters	Species			
	*A. mahidoliae*	*A. sericea*	*P. dasyphylla*	*P. filipes*
**TRANSVERSE LEAF SECTIONS**				
**Petiole pulvinus** (Fig. 3A, E, I, M)				
Outline of petiole pulvinus	Rounded to ovoid	Rounded	Rounded to ovoid	Rounded
Vascular tissue	Collateral type arranged in a single bundle, interspersed with parenchyma <5 pointed	Collateral type arranged in a single bundle, interspersed with parenchyma <5 pointed	Collateral type arranged in a single bundle, interspersed with parenchyma >5 pointed	Collateral type arranged in a single bundle, interspersed with parenchyma >5 pointed
**Petiole** (Fig. 3B, F, J, N)				
Outline of petiole	Rounded to obovoid with two ridges on the adaxial surface	Rounded to obovoid with two ridges on the adaxial surface	Rounded to ovoid with two ridges on the adaxial surface	Rounded to ovoid with two ridges on the adaxial surface
Number of epidermal layers	1	1	1–2	1
Outline of ridges	Obovoid	Obovoid	Rounded to ovoid	Rounded to ovoid with a broad base
Vascular tissue	Collateral type, with two small lateral bundles on ridges and a main, central, continuous collateral bundle arranged in a single arc	Collateral type, with two small lateral bundles on ridges and a main, central, discontinuous collateral bundle arranged in a single arc	Two amphicribral lateral bundles on ridges and a main, central, continuous collateral bundle	Two amphicribral lateral bundles on ridges and a main, central, continuous collateral bundle
**Rachis** (Fig. 3C, G, K, O)				
Outline of rachis	Rounded to obovoid	Rounded to obovoid	Rounded to ovoid	Rounded to ovoid
Number of epidermal layers	1	1	2	1
Outline of ridges	Obovoid	Obovoid	Rounded to ovoid	Rounded to ovoid with a broad base
Vascular tissue	Collateral type, with two small lateral bundles on ridges and a main, central, continuous collateral bundle arranged in a single arc	Collateral type, with two small lateral bundles on ridges and a main, central, discontinuous collateral bundle arranged in a single arc	Two amphicribral lateral bundles on ridges and a main, central, continuous collateral bundle	Two amphicribral lateral bundles on ridges and a main, central, continuous collateral bundle
**Petiolule pulvinus** (Fig. 3D, H, L, P)				
Vascular tissue	Collateral arranged in a single arc	Collateral arranged in a single arc	Collateral arranged in a single arc	Collateral arranged in a single arc
Width of petiolule pulvinus (x axis, µm)	1056.75±191.03	917.32±46.86	1468.18±266.58	1450.81±125.72
Thickness of petiolule pulvinus (y axis, µm)	991.36±184.97	888.60±42.32	1442.86±275.09	1245.15±72.79
**Midrib** (Fig. 4A, D, G, J)				
Outline of midrib	Flattened outline on the adaxial surface with single convexity on the abaxial surface	Flattened outline on the adaxial surface with single convexity on the abaxial surface	Flattened outline on the adaxial surface with single convexity on the abaxial surface	Flattened outline on the adaxial surface with single convexity on the abaxial surface
Number of collenchyma cell layers above the vascular tissue	3–4	3–4	5–6 (indistinct)	5–6 (indistinct)
Vascular tissue	Collateral arranged in a single arc	Collateral arranged in a single arc	Collateral arranged in a single arc	Collateral arranged in a single arc
**Leaflet blade** (Fig. 4B, E, H, K)				
Thickness of leaflet blade (y axis, µm)	143.05±25.85	147.00±23.83	148.65±27.58	141.23±53.96
Number of palisade cell layers	1	1	1	1–2
Presence of idioblasts with twinned kinked crystals	Present	Present	Absent	Absent
**Leaflet margin** (Fig. 4C, F, I, L)				
Outline	Rounded with a straight outline	Rounded with a straight outline	Acute with downward outline	Acute with downward outline

The middle portion of this structure in all species comprises a single-layered epidermis, which is thinly cuticularized in *Afgekia* and thickly cuticularized in *Padbruggea*. In both genera, the vascular bundles were arranged in a prominent circular ring surrounding a central pith composed of parenchymatous tissue (Fig. 3A, E, I, M). In *Padbruggea*, the phloem fibers formed a discontinuous ring near the cortex, often divided into several distinct strands by intervening parenchyma derived from the central pith (Figs 3I, 3M, 8B), a feature less evident in *Afgekia* (Figs 3A, 3E, 8A).

Calcium oxalate crystals were broadly dispersed throughout the vascular tissue and surrounding cortical parenchyma, particularly adjacent to the vascular bundles. This pattern was observed in all four species. Red dark-staining deposits were prominently observed in cortical cells of *A. sericea* and *P. filipes* (Fig. 3E, M), moderately present in *A. mahidoliae* (Fig. 3A), but absent in *P. dasyphylla* (Fig. 3I).

### Petiole

The petiole outline was generally rounded to ovoid or obovoid across all species (Fig. 3B, F, J, N). On the adaxial side, it exhibited a sulcate contour characterized by two prominent ridges separated by a central furrow. In *Afgekia*, the abaxial outline tended to be obovoid (Fig. 3B, F). In contrast, species of *Padbruggea* displayed more uniformly ovoid to rounded outlines with subtly undulating margins (Fig. 3J, N).

The cuticle was notably thicker in *Padbruggea* than in *Afgekia*. *P. dasyphylla* seemed to display bistratified epidermis (Fig. 8C), but it is uncertain whether they represent a double epidermis or an epidermis plus a hypodermis. In contrast, the other species had a single-layered epidermis. The vascular system of the petiole in all examined taxa comprised three distinct bundles: a centrally located main vascular bundle and two smaller accessory bundles positioned at the adaxial ridges of the petiole. Almost all have a closed arc configuration of the main bundle (Fig. 3B, J, N), except *A. sericea* (Fig. 3F). The accessory bundles in *Afgekia* were small, ovoid, and collateral, positioned within the protruding adaxial ridges (Fig. 3B, F). By contrast, those in *Padbruggea* were rounder, amphicribral, and contained a central parenchymatous pith (Figs 3J, 3N, 8E, 8F).

Crystals were widespread, notably concentrated in the parenchymatous cells of the cortex adjacent to the vascular system. These deposits were visible across all taxa and displayed a similar arrangement to that found in the petiole-pulvinus, though with slightly higher density in *Padbruggea*. In contrast to the pattern observed in the petiole-pulvinus, dark-staining deposits within the subepidermal, cortical region of the petiole were detected only in *P. dasyphylla* (Fig. 3J).

### Rachis

The anatomical structure of the rachis in both genera closely resembles that of the petiole (Fig. 3C, G, K, O), comprising a single layer of epidermis in most species, except for *P. dasyphylla*, which seemed to be composed of two layers. A notable difference was observed only in *Afgekia*, where the two adaxial accessory bundles are distinctly separated from each other (Fig. 3C, G), a condition that is particularly evident in *A. sericea*. In contrast, in *Padbruggea* these bundles are positioned much closer together, and in *P. dasyphylla* they may even be partially connected (Fig. 3K). In *A. sericea*, this region is further characterized by the presence of vertically elongated, palisade-like parenchyma cells beneath the epidermis, indicating a transitional anatomical zone toward the leaflet blade (Figs 3G, 8G).

A comparable pattern of crystal deposition was observed. Crystals were present throughout the vascular zone and were particularly dense in the inner cortical parenchyma near the vascular bundles. This pattern was consistent among all examined species. Cortical deposits of dark-staining material were weakly present in *P. filipes* (Fig. 3O) and remained abundant in *P. dasyphylla* (Fig. 3K).

### Petiolule-pulvinus

The anatomical structure of the petiolule pulvinus was generally consistent between the two genera, particularly in the broadly elliptic to rounded outline and the presence of bicollateral vascular tissue arranged in a single arc (Fig. 3D, H, L, P). Nevertheless, only minor but consistent differences were observed between the genera. The epidermis consisted of a single layer of small, rounded cells encircling the surface, with a conspicuously thicker cuticle observed in *Padbruggea*. In addition, both the overall size of the petiolule pulvinus and the size of the vascular bundles were generally smaller in *Afgekia* species than in *Padbruggea* (Table 3).

The cortex in the petiole-petiolule section, particularly in *Padbruggea*, showed abundant crystals, indicating localized enhanced crystal accumulation in this zone. Dark-staining deposits were absent in *P. dasyphylla* (Fig. 3L) but present in *A. sericea*, *A. mahidoliae*, and *P. filipes* (Fig. 3D, H, P).

### Midrib

The midrib outline was largely similar across species, appearing nearly oval to circular on the abaxial side in all taxa examined (Fig. 4A, D, G, J). All taxa exhibited a flattened outline along the adaxial side. A slightly undulated cortical margin was observed on the abaxial side in *A. sericea* and *P. dasyphylla* (Fig. 4D, G).

In all four species, the epidermis was composed of a single layer of cells with a conspicuously thick cuticular layer. Collenchyma cells were situated under the adaxial epidermal layer. The vascular bundle in all species was arranged in a collateral pattern, forming a single arc.

Crystals were concentrated around vascular bundles and, to a lesser extent, in the cortical parenchyma. Among the taxa, *A. mahidoliae* exhibited the highest crystal abundance in this region (Fig. 4A), whereas the other species showed only sparse deposition. Cortical accumulation of stained deposits reappeared in *P. dasyphylla* and *P. filipes* (Fig. 4G, J), while both *Afgekia* species lacked such deposits in this region (Fig. 4A, D).

### Leaflet blade and leaflet margin

All species exhibited a single layer of slightly rectangular to rounded epidermal cells on both adaxial and abaxial surfaces (Fig. 4B, E, H, K). The adaxial epidermis was generally larger and covered by a thicker cuticle, particularly in *Padbruggea* (Fig. 4H, K), which developed a more conspicuous cuticular layer than *Afgekia* (Fig. 4B, E).

All taxa exhibited a dorsiventral mesophyll arrangement. The palisade mesophyll consisted of cylindrical, chloroplast-rich cells oriented perpendicularly to the leaflet surface. *P. filipes* was distinguished by a two-layered palisade tissue (Fig. 4K), while *P. dasyphylla* and *Afgekia* species typically had a single palisade layer (Fig. 4B, E, H). The spongy mesophyll consisted of irregularly shaped, chloroplast-bearing cells. It was loosely arranged in *Afgekia*, but appeared more compact in *Padbruggea*, especially in *P. dasyphylla* (Fig. 4H). The thickness ranged from 36.72–105.24 µm, with *P. dasyphylla* having the thickest layer among the examined taxa (Table 3).

Small calcium oxalate crystals were detected near vascular tissues in all taxa. Crystals were more frequent in *Padbruggea* than in *Afgekia*. Notably, *A. mahidoliae* was distinguished by the presence of idioblast cells containing large solitary crystals prominently located within the palisade parenchyma along the veins (Figs 4B, 8H). Such idioblasts were less frequent in *A. sericea* and absent in both *Padbruggea* species. Moreover, dark-staining deposits were observed in the palisade mesophyll cells of *A. sericea* and *P. filipes* (Fig. 4D–F, J–L), very rarely in *A. mahidoliae* (Fig. 4A–C), and exclusively in the spongy mesophyll of *P. filipes* and *P. dasyphylla* (Fig. 4G–L).

Leaflet margin outlines differed between genera: *Afgekia* displayed rounded margins with an overall straight outline (Fig. 4C, F), whereas *Padbruggea* exhibited slightly acutely angled margins that were gently curved downward (Fig. 4I, L).

### Trichomes

Type, size, density, and distribution of trichomes on various parts of the compound leaf revealed distinct differences and similarities across the studied taxa (Table 4; Figs 5, 6; Suppl. material 1: table SS4). Two major types of trichomes were identified: glandular and eglandular, with both types present in most species examined.

**Table 4.** Comparison of selected leaf micro-morphological characters (see Suppl. material 1: table SS4 for additional characters).

Characters	Species			
	*A. mahidoliae*	*A. sericea*	*P. dasyphylla*	*P. filipes*
**TRICHOMRS**				
**Petiole pulvinus** (Fig. 5A, D, G, J)				
Indumentum on surface	Densely hairy	Densely hairy	Densely hairy	Densely hairy
Types of eglandular trichome	3 types (LS, MS, SS)	3 types (LS, MS, SS)	1 type (SC)	1 type (SC)
Types of glandular trichome	1 type (GI)	1 type (GI)	Absent	Absent
**Petiole** (Fig. 5B, E, H, K)				
Indumentum on surface	Densely hairy	Densely hairy	Densely hairy	Densely hairy
Types of eglandular trichome	3 types (LS, MS, SS)	3 types (LS, MS, SS)	2 types (MC, SC)	2 types (MC, SC)
Types of glandular trichome	1 type (GI)	1 type (GI)	Absent	1 type (GII)
**Rachis** (Fig. 5C, F, I, L)				
Indumentum on surface	Hairy; pubescent and sericeous	Hairy; pubescent and sericeous	Hairy; strigose and tomentose	Hairy; strigose and tomentose
Types of eglandular trichome	3 types (LS, MS, SS)	3 types (LS, MS, SS)	2 types (MC, SC)	2 types (MC, SC)
Types of glandular trichome	1 type (GI)	1 type (GI)	Absent	1 type (GII)
**Petiolule pulvinus** (Fig. 6A, E, I, M)				
Indumentum on surface	Densely hairy	Densely hairy	Densely hairy	Densely hairy
Types of eglandular trichome	3 types (LS, MS, SS)	3 types (LS, MS, SS)	2 types (MC, SC)	3 types (LC, MC, SC)
Types of glandular trichome	1 type (GI)	1 type (GI)	Absent	Absent
**Midrib** (Fig. 6B, C, F, G, J, K, N, O)				
Indumentum on surface	Densely hairy	Densely hairy	Densely hairy	hairy
Types of eglandular trichome	3 types (LS, MS, SS)	3 types (LS, MS, SS)	3 types (LC, MC, SC)	3 types (LC, MC, SC)
Types of glandular trichome	1 type (GI)	2 types (GI and GIII), GIII is restricted to veins	Absent	1 type (GII)
**Leaflet blade**				
**Adaxial surface** (Fig. 1A, D, G, J)				
Indumentum on surface	Hairy	Densely hairy	Glabrescent, occasionally pubescent with hairs restricted to veins	Glabrescent, occasionally pubescent with hairs restricted to veins
Types of eglandular trichome	3 type**s** (LS, MS, SS)	3 type**s** (LS, MS, SS)	1 type (SC)	2 types (MC, SC)
Number of subsidiary cells of eglandular trichomes	5–17	6–8 (rarely 5 or 10)	10–16	6–14
Density of eglandular trichomes (per cm^2^)	180±17.02	386.67±75.06	No density or 290.00±18.03	No density or 30.67±3.06
Types of glandular trichome	1 type (GI)	1 type (GI)	Absent	Absent
**Abaxial surface** (Fig. 2A, D, G, J)				
Indumentum on surface	Hairy	Densely hairy	Glabrescent, occasionally pubescent with hairs restricted to veins	Glabrescent, occasionally pubescent with hairs restricted to veins
Types of eglandular trichome	3 type**s** (LS, MS, SS)	3 type**s** (LS, MS, SS)	2 types (MC, SC)	2 types (MC, SC)
Number of subsidiary cells of eglandular trichomes	5–17	6–8 (rarely 5 or 10)	10–16	6–14
Density of eglandular trichomes (per cm^2^)	610.00±40.00	1590.00±26.46	No density or 316.67±179.19	No density or 94.67±22.74
Types of glandular trichome	1 type (GI)	1 type (GI)	Absent	1 type (GII), restricted to veins
**LEAVES ARCHITECTURE** (Fig. 7)				
Venation orders	6	5	6	6
Primary vein framework	Pinnate	Pinnate	Pinnate	Pinnate
Perimarginal veins	Absent	Absent	Fimbrial vein	Fimbrial vein
Major secondary spacing	Decreasing proximally	Decreasing proximally	Irregular	Irregular
Intersecondary length	<50% of subjacent secondary	<50% of subjacent secondary	Typically, >50% of adjacent secondary; variable	Typically, >50% of adjacent secondary; variable
Intercostal tertiary vein fabric	Mixed percurrent	Mixed percurrent	Absent	Absent
Epimedial tertiary	Mixed percurrent	Mixed percurrent	Absent	Absent
Areolation	Moderate development	Moderate development	Good development	Good development
FEVs branching	Mostly with two or more branches	Mostly with two or more branches	Mostly unbranched	Mostly unbranched
FEVs termination	Simple	Simple	Simple	Simple
Marginal ultimate venation	Incomplete loops	Incomplete loops	Absent	Looped

Among the glandular types, three subtypes were distinguished based on stalk and head morphology (Fig. 8J–L): GI, long capitate glandular trichomes with multicellular, elongated stalks and bowl-shaped heads (Figs 5B, 5C, 5E, 5F, 8J), GII, intermediate capitate glandular trichomes with uni-elongate or multicellular stalks and club-like heads (Fig. 8K), and GIII, short capitate glandular trichomes with short multicellular stalks and round-like heads (Fig. 8L). For eglandular trichomes, six size- and orientation-based bicellular categories were recognized for descriptive purposes: LS, large straight (>1000 μm), MS, medium straight (500–1000 μm), SS, small straight (<500 μm), LC, large curly, MC, medium curly, and SC, small curly (Table 4; Figs 5, 6).

Overall, several trichome types were shared among different parts of the compound leaf, including the leaflet blade, petiole, rachis, and pulvinus, whereas other trichome types showed restricted distributions, being confined to specific leaf regions and/or particular taxa. The GI-type glandular trichomes were widely distributed across all parts of the compound leaf, including the petiole-pulvinus, petiole, rachis, petiolules-pulvinus, midrib, and leaflet blade in all *Afgekia* species. In contrast, GIII-type glandular trichomes showed a restricted distribution, being sparsely confined to the midrib and associated venation in *A. sericea*. Interestingly, *P. filipes* possessed only the GII-type, which was restricted to the petiole, rachis, and midrib.

Straight-type eglandular trichomes (LS, MS, SS) were observed exclusively in *Afgekia* (Figs 5A–F, 6A–H), whereas curly types (SC, MC, LC) were restricted to *Padbruggea* (Figs 5G–L, 6I–P). In *Afgekia*, all three straight trichome types were present across all examined leaf parts. By contrast, *Padbruggea* exhibited curly trichomes in specific leaf regions: SC occurred on the petiole-pulvinus and leaflet blade, MC on the petiole, rachis, and petiolules, and LC was confined to the midrib, where it co-occurred with the other curly types.

Trichome density was consistently higher on the abaxial than the adaxial surface in all taxa. Among the species studied, *A. sericea* had the highest trichome density on both surfaces, whereas *P. filipes* had the lowest (Suppl. material 1: table SS4).

### Leaf architecture

A pinnate primary venation pattern with up to six orders of venation and festooned brochidodromous secondary veins was observed in most species (Fig. 7A–C, G–L), except *A. sericea*, which exhibited only five venation orders (Fig. 6D–F). Since the terminal and lateral leaflets exhibited highly similar architectural features across all species, their characteristics are described collectively. However, certain architectural traits were found to distinguish between *Afgekia* and *Padbruggea* (Table 4; Fig. 7; Suppl. material 1: table SS4).

Perimarginal veins were absent in both *Afgekia* species (Fig. 7C, F), whereas they appeared as fimbrial veins in *Padbruggea* (Fig. 7I, L). Likewise, the secondary vein spacing was consistently decreasing proximally in *Afgekia* (Fig. 7A, D), in contrast to the irregular spacing observed in *Padbruggea* (Fig. 7G, J). Intersecondary veins in *Afgekia* were consistently shorter than 50% of the length of the subjacent secondaries, while in *Padbruggea*, they occasionally exceeded this threshold. Intercostal and epimedial tertiary veins were observed only in *Afgekia*, with a mixed percurrent pattern comprising basiflexed and looped courses (Fig. 7A, C, D, F). These features were absent or not applicable in *Padbruggea*. Areolation was more prominent in *Padbruggea* (Fig. 7G–L) than in *Afgekia* (Fig. 7A–F). In *Afgekia*, simple freely ending ultimate veins (FEVs) were predominantly two- or more-branched (Fig. 7B, E), while in *Padbruggea* they were typically unbranched (Fig. 7H, K). Marginal ultimate venation also varied: *Afgekia* exhibited incomplete loops (Fig. 7B–E), *P. filipes* showed looped patterns (Fig. 7K), and *P. dasyphylla* lacked marginal loops entirely (Fig. 7H).

## Discussion

### Epidermal cells and stomatal characters:

Epidermal traits, particularly the shape and anticlinal wall patterns of epidermal cells, proved stable and taxonomically informative across all taxa studied. In *Afgekia* and *P. dasyphylla*, epidermal cells on both surfaces were consistently undulate to jigsaw-shaped, whereas *P. filipes* displayed polygonal to irregular outlines with nearly straight walls adaxially, contrasting with undulate forms abaxially. These differences, though subtle, were consistent across populations and thus support species-level identification. Such features have been widely recognized for their diagnostic value in Fabaceae and other angiosperm families (Metcalfe and Chalk 1950, 1979; Yang and Lin 2005; Zou et al. 2008; Devecchi et al. 2014; Silva et al. 2018; Zhao et al. 2022b)

While qualitative traits like wall sinuosity were reliable, variation in epidermal cell size between surfaces was also observed. This quantitative variation may reflect genetic and developmental factors, such as ploidy level and genome size, as well as ecological conditions like light intensity and moisture (Kondorosi et al. 2000; Mizukami 2001). Notably, sinuous walls are generally linked to mesic environments, while straighter walls correlate with xeric habitats (Stace 1965; Gifford and Foster 1989; Bidhendi et al. 2019). However, the present data challenge this correlation: *Afgekia*, occurring in drier, high-light sites, had more sinuous cell walls than *Padbruggea*, which occupies moist, shaded habitats. Similar ecological inconsistencies have been reported elsewhere, highlighting that these anatomical traits are more closely tied to phylogeny than environment (Song et al. 2020; Zhao et al. 2022b).

Stomatal type and distribution also provided meaningful distinctions. All taxa were hypostomatic, except *P. filipes*, which was amphistomatic and exhibited stomatal presence even along the midrib – a rare condition in the group. *Afgekia* displayed both paracytic and anomocytic stomata, while *Padbruggea* had exclusively anomocytic types. The coexistence of multiple stomatal types in *Afgekia* aligns with prior findings that stomatal morphology can serve as a stable character at inter- and infrageneric levels (Metcalfe and Chalk 1979; Zou et al. 2008; Devecchi et al. 2014; Silva et al. 2018; Song et al. 2020; Zhao et al. 2022b). Furthermore, epidermal features of *Padbruggea* closely resemble those reported in *Wisteria sinensis* (Sims) DC. (Demeshko et al. 2022), the only other Wisterieae member with published epidermal data, suggesting a conserved morphological pattern within the tribe. This supports the evolutionary proximity between *Padbruggea* and *Wisteria*, as previously proposed by Compton et al. (2019) (Suppl. material 2) and Duan et al. (2021b).

Stomatal density (SD) and stomatal index (SI) varied among species, with *Padbruggea* generally showing higher SD than *Afgekia*. Intriguingly, *A. sericea* had the highest SI, while *P. filipes* recorded the lowest. Although SD and SI are known to fluctuate with environmental factors such as CO_2_ concentration, humidity, and irradiance (Metcalfe and Chalk 1950; Royer 2001). The consistent interspecific differences in this study suggest their potential taxonomic utility when considered alongside stable qualitative characters (Metcalfe and Chalk 1979; Dickison 2000). From an ecological perspective (Dickison 2000; Aasamaa et al. 2001; Hetherington and Woodward 2003; Casson et al. 2009; Hamanishi et al. 2012; Lawson and Blatt 2014), lower stomatal density, as found in *Afgekia*, may reflect xeromorphic adaptations. Plants in dry or high-light habitats (including limestone substrates, as in *A. mahidoliae*) often exhibit fewer but larger stomata, promoting water-use efficiency and tighter regulation of gas exchange. This contrasts with higher stomatal densities typically associated with shaded or humid environments. The observed trends reinforce the functional and phylogenetic relevance of stomatal traits in species differentiation within this group.

Epidermal cell patterns and stomatal characters have long been recognized as taxonomically informative across diverse angiosperm lineages beyond Fabaceae. Variations in epidermal cells have been successfully applied in species and generic delimitation in families such as Anacardiaceae (Tipmontiane et al., 2018), Convolvulaceae (Traiperm et al., 2017), and Zingiberaceae (Zhao et al., 2022a), where these traits are considered relatively stable and under strong genetic control. The consistency of these characters observed in *Afgekia* and *Padbruggea* therefore supports their broader taxonomic relevance and reinforces their utility for species delimitation.

### Petiole/ petiolule-pulvinus

Comparisons of the petiole-pulvinus and petiolule-pulvinus revealed a broadly conserved vascular framework across *Afgekia* and *Padbruggea*, but also highlighted subtle, taxonomically informative differences. Both genera shared the typical Fabaceae arrangement of Collateral vascular bundles in a single arc with or without a central parenchymatous pith, with a rounded to slightly elliptical organ outline. This configuration mirrors the petiolule-pulvinus anatomy described in *Nanhaia speciosa* (Ong et al. 2024), the only other Wisterieae species with comparable data, and is consistent with general patterns in Fabaceae (Metcalfe and Chalk 1979; Silva et al. 2022). Despite the overall similarity, *Padbruggea* exhibited a more complex internal differentiation on the petiole-pulvinus: its phloem fibers often formed a discontinuous peripheral ring interrupted by rays of parenchyma derived from the pith, structures reduced or absent in *Afgekia*. Relative bundle sizes also varied, with *A. mahidoliae* and *P. filipes* showing proportionally larger vascular bundles than *A. sericea* and *P. dasyphylla*.

### Petiole and rachis

The petiole and rachis followed the Fabaceae norm of three main vascular bundles: one main, large, arc-shaped central bundle, positioned slightly toward the abaxial side and two smaller accessory bundles adaxially (Matos et al. 2020; Rajput et al. 2021; Thacker et al. 2021; Souto et al. 2024. However, morphological distinctions between taxa were evident in both outline and internal structure. Petiole cross-sections in *Afgekia* tended to have obovoid outlines, while *Padbruggea* displayed more rounded forms. Notably, a structure resembling two epidermal-like layers was observed in the petiole and rachis of *P. dasyphylla*, distinguishing it from its congener *P. filipes*. Double-layered or multiseriate epidermis is uncommon in Fabaceae, occurring only sporadically in organs such as stems, leaves, or pods, and often associated with xeromorphic or arid-environment adaptations (Chauhan and Pandey 2014; Coutinho et al. 2016; Aguilar-Rodríguez et al. 2019; Varilla González et al. 2023). In *P. dasyphylla*, however, the petiole and rachis exhibited what appeared to be a biseriate epidermal structure, in which the outer and inner layers consisted of cells that were nearly identical. This made it unclear whether the condition represented a true double epidermis or an epidermis underlain by a hypodermis. Moreover, Coutinho et al. (2016) reported the presence of secretory idioblasts in the epidermis of *Chamaecrista* sect. *Apoucouita*, noting that these idioblasts sometimes developed a thinner periclinal cell wall that effectively partitioned the cell, giving the epidermis the appearance of two layers. Resolving the nature of this characteristic in *P. dasyphylla* will require further ontogenetic investigation.

Accessory bundles also differed in both type and placement. In *Afgekia*, they were small, ovoid, collateral bundles embedded within the adaxial ridges, whereas in *Padbruggea* they were larger, amphicribral bundles with a central pith. In addition, the two adaxial ridges of the rachis in *Afgekia* were more extended and sometimes formed prominent protuberances (as in *A. sericea*) that enclosed the accessory vascular bundles, contrasting with the more compact and rounded ridge configuration in *Padbruggea*. These differences suggest that intermediate organs such as the rachis, often overlooked, can provide critical taxonomic clues.

### Midrib, leaflet blade, and leaflet margin

The anatomical structure of the leaflet midrib, blade, and margin demonstrated consistent generic differences with potential taxonomic significance, while also reflecting varying degrees of ecological adaptation. Despite sharing a common vascular pattern, collateral bundles are arranged in a single arc. All taxa exhibited distinct morphological traits.

Midrib outlines were generally nearly circular to oval in the abaxial side, with *P. filipes* showing a more oval shape, *A. sericea* and *P. dasyphylla* uniquely possessing a slightly undulated cortical outline. Although *Padbruggea* species developed notably thicker midribs, *Afgekia* taxa exhibited smaller but structurally similar configurations. All species featured a single-layered epidermis with a thick cuticle and subepidermal collenchyma, typical features of Fabaceae (Metcalfe and Chalk 1979; Devecchi et al. 2014; Silva et al. 2018; Ong et al. 2024).

In the leaflet blade, all species shared a dorsiventral mesophyll arrangement. However, interspecific and intergeneric differences emerged in tissue stratification and compactness. The presence of a two-layered palisade mesophyll in *P. filipes*, in contrast to the single-layered structure in other taxa, suggests enhanced photosynthetic capacity, a trait usually associated with exposure to high light intensity and low air humidity (Dickison 2000; Torre 2003). Paradoxically, *Padbruggea* species usually grow in more shaded and moist environments than those of *Afgekia*, implying that such internal structure may have been retained through evolutionary history rather than being strictly environmentally induced.

Leaflet margin profiles provided further distinguishing features: *Afgekia* species possessed rounded margins with straight or gently curved outlines, whereas *Padbruggea* exhibited sharply acute, slightly revolute margins. These traits, though subtle, proved stable across populations and reinforce their value for taxonomic delimitation.

### Trichomes, calcium oxalate crystals, and dark-staining deposits

Trichome morphology and distribution exhibited clear intergeneric differences. Bearing three types of glandular trichomes (GI, GII, and GIII) and six types of eglandular trichomes (LS, MS, SS, LC, MC, and SC). *Afgekia* showed greater diversity; GI-type glandular trichomes were consistently present across all leaf parts, while GIII-type was found only in *A. sericea*. In contrast, *Padbruggea* possessed only the GII-type, confined to vascular-rich zones (petiole, rachis, midrib, and along veins). Eglandular trichomes also varied between genera: straight forms (LS-SS) were unique to *Afgekia*, whereas curly forms (SC-LC) characterized *Padbruggea*. These combinations of presence, type, and distribution reflect strong taxonomic signals at both genus and species levels (Uphof 1962; Stenglein et al. 2003; Chukwuma et al. 2014).

Interspecific variation was also apparent in eglandular trichome density, especially on the adaxial leaflet surface. *Afgekia. sericea* and *P. dasyphylla* exhibited dense coverage, while *A. mahidoliae* and *P. filipes* had much sparser trichomes. Notably, these density patterns were consistent across populations, supporting their diagnostic value. Similarly, the specific occurrence of glandular trichome types in different taxa reinforces their relevance for species delimitation.

Functionally, eglandular trichomes may contribute to adaptation under high light or arid conditions by reflecting solar radiation, limiting transpiration, and deterring herbivores and pathogens (Liakoura et al. 1997; Wagner et al. 2004) – roles likely relevant in *A. sericea*. However, in *P. dasyphylla*, this function may be limited, as many trichomes abscise upon leaf maturation. Glandular trichomes, often located along the midrib and minor veins, are thought to secrete defensive compounds and protect key transport tissues (Wagner et al. 2004; LoPresti 2016; Murungi et al. 2016; Tozin and Rodrigues 2017; Li et al. 2018), though their effectiveness may diminish with age in *Padbruggea* species. It is worth noting that this study focused exclusively on the presence, absence, and morphology of trichomes for taxonomic purposes. Functional interpretations, while plausible, remain speculative and should be tested through targeted physiological and ecological studies.

Trichome morphology and distribution are likewise widely used as diagnostic characters in plant taxonomy across multiple angiosperm families. Differences in trichome type, size, orientation, and density have been shown to provide reliable taxonomic signals in groups such as Asteraceae (Liesenfeld et al. 2019), Fagaceae (Fortini et al. 2009), and Solanaceae (Watts and Kariyat 2021). The clear intergeneric and interspecific differences in trichome types and distribution patterns documented in this study are therefore consistent with their recognized taxonomic value in other plant lineages.

Calcium oxalate crystals were consistently observed in all studied species of *Afgekia* and *Padbruggea*, with a typical localization around vascular tissues and adjacent cortical parenchyma across most vegetative leaf organs. This deposition pattern corresponds with earlier reports in many families, where crystals, especially prismatic forms, are typically found near vascular bundles or their sheaths (Zindler-Frank et al. 2001; Nakata 2002; Lersten and Horner 2011).

Despite this general similarity, diagnostic differences were evident. *Afgekia* species displayed distinctive idioblasts containing large solitary crystals, especially within the palisade parenchyma – a feature absent in *Padbruggea*. Additionally, the density of crystal deposits varied between species, allowing discrimination between *A. mahidoliae* and *A. sericea*, further reinforcing the taxonomic utility of these structures. As *A. mahidoliae* is a limestone-adapted species typically found in or near limestone habitats, whereas *A. sericea* is not, it is unsurprising that crystal deposition in the leaves of *A. mahidoliae* was higher in this study. Remarkably, this characteristic persisted even though the sampled plants were cultivated as ornamentals far from their natural limestone habitats. The occurrence of idioblasts has been recognized as a taxonomically informative trait, often reflecting phylogenetic patterns more reliably than environmental variables (Lersten and Horner 2004; Franceschi and Nakata 2005). Their distribution is under strong genetic control, which may explain the stability of crystal localization across populations in this study despite ecological variation.

Beyond taxonomy, calcium oxalate crystals exhibit functional versatility. They may act as localized calcium sinks, contribute to mechanical support, or serve as anti-herbivore defenses (Molano-Flores 2001; Franceschi and Nakata 2005; Korth et al. 2006). In particular, idioblasts in the leaf lamina, such as those observed in *Afgekia*, may enhance tissue rigidity, reflect light into deeper photosynthetic layers, or facilitate water transport and storage (Karabourniotis 1998; Cutler 2005; Brodribb et al. 2010). The prominent crystal accumulation in *Padbruggea*, especially within the petiole-petiolule and midrib, may reflect lineage-specific strategies for calcium regulation or physiological buffering, although functional verification would require further study.

Dark-staining deposits, presumably phenolic or tannin-related compounds as commonly reported in several group plants (De-Luna et al. 2014; Neves et al. 2016; Raman et al. 2018; Naik and Sellappan 2024), also exhibited systematic variation across taxa. In *Afgekia*, these deposits were prominent in *A. sericea* and moderate in *A. mahidoliae*, while in *Padbruggea*, the pattern was more variable, with *P. filipes* showing deposits in multiple leaf parts and *P. dasyphylla* only in select regions. These secretory elements, potentially associated with defensive functions, show remarkable consistency across all populations within a species, suggesting their potential utility in taxonomic differentiation.

### Leaf architecture

Although all taxa shared a pinnate primary venation framework and festooned brochidodromous secondary veins, common characteristics among Papilionoideae (Luckow 2002; Ellis et al. 2009; Devecchi et al. 2014; Iglesias et al. 2021), the presence of only five orders of venation in *A. sericea*, compared to six in the other species, demonstrates interspecific variation in venation hierarchy. A higher number of venation orders is functionally associated with increased hydraulic redundancy, mechanical support, and protection against herbivory (Zwieniecki et al. 2002; Sack et al. 2004; Mendez-Alonzo et al. 2013).

Perimarginal veins further differentiated the genera: *Padbruggea* displayed fimbrial veins, whereas *Afgekia* lacked perimarginal venation. This pattern aligns with observations that perimarginal vein presence is frequently genus-specific and reflects phylogenetic divergence (Cardoso and Sajo 2006; Ellis et al. 2009). Similarly, secondary vein spacing was consistently proximally decreasing in *Afgekia* but irregular in *Padbruggea*. Additional characters, including intersecondary vein length (consistently short in *Afgekia* vs. occasionally >50% in *Padbruggea*), presence of intercostal and epimedial tertiary veins (restricted to *Afgekia*), and patterns of marginal ultimate venation, reinforce the anatomical distinction between the genera. The absence of marginal loops in *P. dasyphylla*, looped pattern in *P. filipes*, and incomplete loops in *Afgekia* support the taxonomic separation.

Although all observed FEVs in the four studied species were of the simple type, *Afgekia* specimens, particularly *A. mahidoliae*, exhibited large crystal deposits along minor veins that form a network throughout the lamina. This feature may suggest a possible adaptive role under xeric conditions, analogous to tracheoid idioblasts reported in other taxa (Haberlandt 1914; Tucker 1964; Lester and Carvey 1974; Dickison 2000; Luckow 2002). Nonetheless, the presence of idioblasts, when observed, tends to be constant regardless of environmental conditions. Further investigation is required to evaluate the phylogenetic significance of these idioblastic features. Such venation characteristics, when integrated with other morphological and anatomical data, have proven valuable in resolving taxonomic boundaries across various plant families where generic delimitations remain challenging (Cardoso and Sajo 2006; De-Carvalho 2008; Andres-Hernandez and Terrazas 2009; Devecchi et al. 2014).

Taken together, the integrative assessment of leaf architectural, anatomical, and micromorphological traits presented herein not only highlights clear interspecific and intergeneric differentiation between *Afgekia* and *Padbruggea*, but also reinforces the consistency and diagnostic value of these characters across populations. In order to facilitate practical identification of the studied taxa and to synthesize the distinguishing features established in this study, a bracketed key is provided below. This key incorporates the most taxonomically informative characters from multiple leaf traits and reflects the morphological evidence supporting the current taxonomic treatment of *P. filipes* as distinct from *Afgekia*.

### Key to genera and species of *Afgekia* and *Padbruggea*

GI–GIII denote capitate glandular trichomes differing in stalk length (long, intermediate, short) and head shape (bowl-shaped, club-like, round-like), whereas LS–SC denote eglandular trichomes classified by size (large, medium, small) and orientation (straight or curly).

**Table:** 

1	Leaf venation lacking fimbrial veins; major secondary veins proximally decreasing in spacing; intercostal and epimedial tertiary veins mixed percurrent; exmedial course parallel to intercostal tertiary; exterior course basiflexed; areolation moderately developed. Trichomes: straight eglandular types (LS, MS, SS) present; GI-type glandular trichomes present on all leaf parts. Epidermis and stomata: both paracytic and anomocytic stomata present. Leaf transverse section: petiole-pulvinus indistinct or without parenchyma rays interrupting vascular bundles; petiole and rachis with prominent club-shaped adaxial ridges, collateral accessory bundles present; midrib with 3–4 collenchyma layers under epidermis; idioblasts with twinned kinked crystals in palisade parenchyma present; leaf margin rounded with straight outline	**2 (*Afgekia* )**
–	Leaf venation with well-developed perimarginal (fimbrial) veins; major secondary veins irregularly spaced; intercostal and epimedial tertiary veins absent; exmedial course absent; exterior course absent; areolation well-developed. Trichomes: curly eglandular types (LC, MC, SC) present; GI-type glandular trichomes absent. Epidermis and stomata: only anomocytic stomata present. Leaf transverse section: petiole-pulvinus with parenchyma rays between vascular bundles; petiole and rachis with short adaxial ridges and amphicribral accessory bundles; midrib with 5–6 collenchyma layers under epidermis; idioblasts absent; leaflet margin acutely angled with slightly revolute outline	**3 (*Padbruggea* )**
2	Leaf venation with six orders; both leaf surfaces sparsely pubescent; Trichomes: GIII-type glandular trichomes absent; few trichomes present on lamina and veins. Stomatal density <150 stomata/mm^2^. Leaf transverse section: petiole-pulvinus rounded to ovoid; midrib with smooth cortical outline; idioblasts densely distributed along minor veins	***A. mahidoliae***
–	Leaf venation with five orders; both leaf surfaces densely pubescent; Trichomes: GIII-type glandular trichomes present on midrib and main veins. Stomatal density >150 stomata/mm^2^. Leaf transverse section: petiole-pulvinus rounded; midrib with slightly undulate cortical outline; idioblasts sparsely distributed along minor veins	***A. sericea***
3	Venation pattern: major secondary veins attached excurrently; Trichomes: both leaf surfaces densely covered with eglandular trichomes; GII-type glandular trichomes absent. Epidermis and stomata: adaxial epidermis with jigsaw puzzle-shaped anticlinal walls. Stomata hypostomatic (present only on abaxial side). Leaf transverse section: petiole-pulvinus rounded to ovoid; petiole and rachis with two-layered epidermis; palisade mesophyll single-layered	***P. dasyphylla***
–	Venation pattern: major secondary veins both excurrent and basally decurrent; Trichomes: both leaf surfaces sparsely pubescent with eglandular trichomes; GII-type glandular trichomes restricted to petiole, rachis, and veins. Epidermis and stomata: adaxial epidermis polygonal with straight anticlinal walls. Stomata amphistomatic (present on both surfaces). Leaf transverse section: petiole-pulvinus rounded; petiole and rachis with single-layered epidermis; palisade mesophyll single- to double-layered	***P. filipes***

## Conclusion

This study demonstrates the taxonomic utility of integrating multiple anatomical traits, including leaf architecture, trichome morphology, epidermal and stomatal characteristics, and leaf transverse section features, in distinguishing species of *Afgekia* and *Padbruggea*. The consistent inter- and infrageneric variation observed across these traits underscores their value in species delimitation, even when some characteristics appear ecologically contradictory.

The re-evaluation of *Padbruggea filipes*, previously included within *Afgekia*, highlights the strength of integrated anatomical evidence in clarifying generic boundaries. Although *P. filipes* shares some superficial vegetative similarities with *Afgekia*, it exhibits a distinct and consistent set of leaf anatomical characters that clearly supports its separation at the generic level. This anatomical differentiation is congruent with prior phylogenetic evidence (See in Suppl. material 2), reinforcing the value of leaf anatomical traits as reliable indicators of evolutionary divergence.

This anatomical differentiation is congruent with prior phylogenetic evidence, reinforcing the value of leaf anatomical traits as reliable indicators of evolutionary divergence.

Notably, traits such as epidermal cell shape, trichome type, and idioblast distribution were stable across populations, indicating strong genetic control and taxonomic relevance. While environmental factors may influence certain quantitative features, their consistent expression within species validates their diagnostic significance.

Moreover, the detailed comparative analysis contributes to the broader understanding of trait evolution in Wisterieae. It emphasizes the importance of micro-morphological and anatomical characters not only for taxonomy but also for interpreting ecological adaptations and evolutionary trajectories within Fabaceae. Ultimately, the integration of diverse leaf traits provides a powerful framework for resolving complex taxonomic questions and refining generic and species-level delimitations in morphologically similar lineages.

## Additional information

### Use of AI

No use of AI was reported.

### Author ORCIDs

Punvarit Boonprajan https://orcid.org/0009-0006-7251-1928

Yotsawate Sirichamorn https://orcid.org/0000-0002-3026-3894

### Data availability

All of the data that support the findings of this study are available in the main text or Supplementary Information.

## Supplementary materials

Supplementary material 1 Supplementary tables Data type pdf Explanation note **table SS1**. Comparative overview of species assignments to *Afgekia* and *Padbruggea* in major taxonomic treatments from 1984 to the present, following the framework of Compton et al. (2019). **table SS2**. Comparison of leaf epidermal characters. **table SS3**. Comparison of leaf transverse section characters. **table SS4**. Comparison of leaf micro-morphological characters. https://binary.pensoft.net/file/1532710This dataset is made available under the Open Database License (http://opendatacommons.org/licenses/odbl/1.0/). The Open Database License (ODbL) is a license agreement intended to allow users to freely share, modify, and use this Dataset while maintaining this same freedom for others, provided that the original source and author(s) are credited. Punvarit Boonprajan, Yotsawate Sirichamorn

Supplementary material 2 Phylogenetic placement of *Afgekia* (pink) and *Padbruggea* (blue) within the tribe Wisterieae, based on Compton et al. (2019) Data type pdf https://binary.pensoft.net/file/1532711This dataset is made available under the Open Database License (http://opendatacommons.org/licenses/odbl/1.0/). The Open Database License (ODbL) is a license agreement intended to allow users to freely share, modify, and use this Dataset while maintaining this same freedom for others, provided that the original source and author(s) are credited. Punvarit Boonprajan, Yotsawate Sirichamorn
