# Supplementary material for: Integration of leaf traits supports the current circumscription of Afgekia Craib. and Padbruggea Miq. (Fabaceae, Wisterieae)
Source: PhytoKeys. 2026 Feb 5;270:289–323. doi: 10.3897/phytokeys.270.181424 (PMC12902768; doi:10.3897/phytokeys.270.181424)
Supplement: Supplementary material 1 — Supplementary tables [file phytokeys-270-289_article-181424__-s001.pdf]

Table S1. Comparative overview of species assignments to *Afgekia* and *Padbruggea* in major taxonomic treatments from 1984 to the present, following the framework of Compton et al. (2019).

Genus	Geesink (1984)	Lôc (1996)	Wei et al. (2010)	Compton et al. (2019)	Duan et al. 2021a, 2021b
Afgekia	*A. filipes*<br>*A. mahidoliae*<br>*A. sericea*	*A. filipes*<br>*A. mahidoliae*<br>*A. sericea*	*A. filipes*<br>-<br>-	-<br>*A. mahidoliae*<br>*A. sericea*	-<br>-<br>*A. sericea*
Padbruggea	-<br>-<br>-	-<br>-<br>-	-<br>-<br>-	*P. filipes*<br>*P. dasyphylla*<br>*P. maingayi*	*P. filipes*<br>*P. dasyphylla*<br>-

Table S2. Comparison of leaf epidermal characters.

**Characters**	**Species**			
	A. mahidoliae	***A. sericea***	***P. dasyphylla***	***P. filipes***
**Adaxial surface** (Fig. 1)				
Width of epidermal cell (µm)	24.52±7.84	30.19±6.61	20.47±5.03	25.67±5.02
Length of epidermal cell (µm)	53.52±6.53	55.35±5.82	36.35±5.66	38.75±7.37
Ratio of epidermal cell size (w : l)	0.47±0.17	0.55±0.12	0.58±0.16	0.68±0.16
**Abaxial surface** (Fig. 2)				
Width of epidermal cell (µm)	31.11±5.88	26.61±7.67	15.02±2.88	16.12±3.84
Length of epidermal cell (µm)	60.98±7.47	40.90±6.47	30.25±6.09	27.93±5.82
Ratio of epidermal cell size (w : l)	0.52±0.12	0.53±0.13	0.52±0.15	0.58±0.12
Width of guard cell (µm)	9.00±1.01	8.36±0.66	6.46±5.15	6.63±0.74
Length of guard cell (µm)	24.01±2.10	21.01±1.13	18.45±7.87	22.24±2.83
Ratio of guard cell size (w : l)	0.38±0.05	0.40±0.04	0.35±0.17	0.29±0.04

Table S3. Comparison of leaf transverse section characters.

Characters	Species			
	A. mahidoliae	*A. sericea*	*P. dasyphylla*	*P. filipes*
**TRANSVERSE LEAF SECTIONS**				
**Petiole pulvinus** (Fig. 3A, E, I, M)				
Width of petiole pulvinus (x axis, μm)	2759.94±121.83	3111.10±973.67	3488.20±571.47	4485.20±684.08
Thickness of petiole pulvinus (y axis, μm)	3264.73±189.83	3364.33±507.84	4076.30±501.37	4680.48±827.64
Ratio of petiole pulvinus size (x : y)	0.85±0.02	0.93±0.05	0.85±0.05	0.96±0.03
Number of epidermal layers	1	1	1	1
Width of vascular tissue (x axis, μm)	1201.03±78.58	999.38±304.08	1449.44±242.68	2135.05±418.01
Height of vascular tissue (y axis, μm)	1318.12±73.45	1052.61±339.90	1449.99±220.17	2278.76±482.14
Ratio of vascular tissue size (x : y)	0.91±0.02	0.96±0.07	1.00±0.04	0.94±0.02
**Petiole** (Fig. 3B, F, J, N)				
Width of petiole (x axis, μm)	1665.90±33.88	1393.39±210.21	1664.69±315.83	1755.13±76.83
Thickness of petiole (y axis, μm)	2108.97±19.83	1738.76±184.57	1923.20±296.70	2061.91±251.55
Ratio of petiole size (x : y)	0.79±0.02	0.80±0.03	0.86±0.07	0.86±0.08
Width of ridges (x axis, μm)	210.26±30.07	356.56±59.65	255.95±74.25	316.33±25.50
Height of ridges (y axis, μm)	199.74±13.81	210.82±62.74	155.96±42.32	225.64±6.99
Ratio of ridges size (x : y)	1.06±0.20	1.77±0.34	1.30±0.11	1.40±0.09
Width of vascular tissue (x axis, μm)	1510.34±35.74	1181.15±261.77	1532.22±308.55	1602.97±60.62
Height of vascular tissue (y axis, μm)	1894.87±72.43	1575.71±137.71	1681.77±285.03	1735.54±124.42
Ratio of vascular tissue size (x : y)	0.80±0.02	0.75±0.10	0.91±0.05	0.93±0.04
**Rachis** (Fig. 3C, G, K, O)				
Width of rachis (x axis, μm)	1373.36±66.99	1291.91±109.61	1513.63±89.99	1318.62±118.58
Thickness of rachis (y axis, μm)	1588.24±76.16	1579.86±129.13	1714.65±157.14	1479.58±145.47
Ratio of rachis size (x : y)	0.87±0.03	0.82±0.02	0.89±0.05	0.89±0.03
Width of ridges (x axis, μm)	98.58±23.98	147.61±16.32	345.19±129.16	262.51±60.93
Height of ridges (y axis, μm)	106.99±13.26	253.83±56.34	246.67±94.70	198.48±8.69
Ratio of ridges size (x : y)	0.91±0.13	0.60±0.11	1.43±0.29	1.33±0.36
Width of vascular tissue (x axis, μm)	1249.19±69.20	1243.75±122.43	1365.42±93.09	1010.29±413.51
Height of vascular tissue (y axis, μm)	1413.27±82.58	1466.20±135.44	1475.12±139.79	1321.41±115.45
Ratio of vascular tissue size (x : y)	0.89±0.05	0.85±0.02	0.93±0.04	0.77±0.32

Characters	Species			
	A. mahidoliae	*A. sericea*	*P. dasyphylla*	*P. filipes*
**Petiolule pulvinus** (Fig. 3D, H, L, P)				
Outline of petiolule pulvinus	Rounded	Rounded	Rounded	Rounded
Ratio of petiolule pulvinus size (x : y)	1.07±0.03	1.03±0.01	1.02±0.09	1.16±0.05
Number of epidermal layers	1	1	1	1
Width of vascular tissue (x axis, µm)	405.61±99.72	314.38±30.10	602.27±120.30	627.68±56.42
Height of vascular tissue (y axis, µm)	304.45±96.67	213.22±36.23	443.33±133.85	421.95±51.46
Ratio of vascular tissue size (x : y)	1.36±0.12	1.49±0.10	1.39±0.15	1.49±0.06
**Midrib** (Fig. 4A, D, G, J)				
Width of midrib (x axis, µm)	509.46±159.14	411.08±7.74	676.57±125.94	627.69±85.17
Thickness of midrib (y axis, µm)	583.85±147.16	521.85±15.34	658.34±84.84	578.24±101.10
Ratio of midrib size (x : y)	0.86±0.09	0.79±0.01	1.04±0.10	1.10±0.11
Number of epidermal layers	1	1	1	1
Width of vascular tissue (x axis, µm)	287.13±101.01	262.00±6.20	566.59±123.88	542.46±55.37
Height of vascular tissue (y axis, µm)	333.54±72.33	322.74±5.28	452.63±73.04	389.32±39.31
Ratio of vascular tissue size (x : y)	0.85±0.14	0.81±0.01	1.25±0.18	1.40±0.11
**Leaf blade** (Fig. 4B, E, H, K)				
Number of epidermal layers	1	1	1	1
Width of adaxial epidermal cell (x axis, µm)	22.81±8.80	21.77±5.28	23.69±7.83	25.04±8.00
Height of adaxial epidermal cell (y axis, µm)	16.37±5.34	13.72±1.97	17.92±3.08	15.54±3.53
Ratio of adaxial epidermal cell size (x : y)	1.54±0.33	1.58±0.23	1.40±0.49	1.63±0.43
Width of abaxial epidermal cell (x axis, µm)	14.80±5.08	20.42±3.41	15.84±3.76	15.38±1.91
Height of abaxial epidermal cell (y axis, µm)	12.47±2.57	13.33±2.38	8.28±2.11	8.68±1.53
Ratio of abaxial epidermal cell size (x : y)	1.16±0.18	1.55±0.27	2.00±0.72	1.84±0.52
Thickness of palisade mesophyll layers (µm)	61.55±11.54	54.43±9.08	49.41±13.53	63.33±20.01
Thickness of spongy mesophyll layers (µm)	45.90±7.07	48.94±6.05	65.41±24.25	50.12±23.95
**Leaf margin** (Fig. 4C, F, I, L)				
Number of epidermal layers	1	1	1	1

Table S4. Comparison of leaf micro-morphological characters.

Characters	Species			
	A. mahidoliae	*A. sericea*	*P. dasyphylla*	*P. filipes*
**TRICHOMRS**				
**Petiole pulvinus** (Fig. 5A, D, G, J)				
Length of eglandular trichomes (µm)	LS = 1482.87±281.37<br>MS = 695.07±178.87<br>SS = 117.50±73.18	LS = 1556.74±332.12<br>MS = 698.72±186.17<br>SS = 166.92±171.59	SC = 403.85±147.45	SC = 320.26±156.64
Length of glandular trichomes (µm)	GI = 396.96±102.77	GI = 390.72±114.76	-	-
**Petiole** (Fig. 5B, E, H, K)				
Length of eglandular trichomes (µm)	LS = 2037.68±606.32<br>MS = 680.68±129.61<br>SS = 329.85±50.84	LS = 2157.76±663.72<br>MS = 676.63±117.07<br>SS = 322.04±36.83	MC = 572.77±38.21<br>SC = 210.16±38.97	MC = 671.39±84.37<br>SC = 206.40±117.81
Length of glandular trichomes (µm)	GI = 531.42±108.38	GI = 537.89±111.64	-	GII = 162.25±79.30
**Rachis** (Fig. 5C, F, I, L)				
Length of eglandular trichomes (µm)	LS = 2179.65±1279.82<br>MS = 702.34±67.57<br>SS = 267.01±88.65	LS = 1059.16±52.51<br>MS = 764.10±140.12<br>SS = 313.79±75.13	MC = 520.31±23.56<br>SC = 362.24±93.19	MC = 553.37±60.66<br>SC = 277.96±74.93
Length of glandular trichomes (µm)	GI = 331.12±205.52	GI = 509.12±47.24	-	GII = 118.95±28.65
**Petiolule pulvinus** (Fig. 6A, E, I, M)				
Length of eglandular trichomes (µm)	LS = 248.21±91.95<br>MS = 658.94±43.09<br>SS = 1735.65±507.15	LS = 275.21±71.29<br>MS = 711.94±79.33<br>SS = 2146.05±571.19	MC = 757.17±148.49<br>SC = 285.98±67.75	LC = 1127.50±218.91<br>MC = 772.02±151.85<br>SC = 360.15±106.77
Length of glandular trichomes (µm)	GI = 323.32±211.59	GI = 321.12±264.81	-	-
**Midrib** (Fig. 6B, C, F, G, J, K, N, O)				
Length of eglandular trichomes (µm)	LS = 2014.28±590.50<br>MS = 612.24±103.42<br>SS = 305.67±142.66	LS = 1388.65±183.04<br>MS = 754.79±104.03<br>SS = 327.89±169.73	LC = 1284.24±284.05<br>MC = 529.08±273.01<br>SC = 434.18±46.04	LC = 1286.02±256.91<br>MC = 627.67±147.02<br>SC = 338.73±35.58
Length of glandular trichomes (µm)	GI = 427.21±106.20	GI = 349.37±108.93<br>GIII = 29.26±4.35	-	GII = 161.50±76.72
**Leaf blade**				
**Adaxial surface** (Fig. 1A, D, G, J)				
Length of eglandular trichomes (µm)	LS = 1314.77±195.80<br>MS = 618.29±122.19	LS = 1093.29±109.90<br>MS = 719.36±127.11	SC = 306.94±113.22	MC = 716.58±8.40<br>SC = 333.20±102.73

**Characters**	**Species**			
	A. mahidoliae	***A. sericea***	***P. dasyphylla***	***P. filipes***
	SS = 205.15±70.53	SS = 255.46±95.99		
Length of glandular trichomes (µm)	GI = 307.59±195.53	GI = 333.34±170.72	-	-
**Abaxial surface** (Fig. 2A, D, G, J)				
Length of eglandular trichomes (µm)	LS = 1456.16±331.23<br>MS = 708.40±121.87<br>SS = 241.80±66.99	LS = 1131.83±110.65<br>MS = 762.27±128.26<br>SS = 292.06±99.18	MC = 600.44±108.13<br>SC = 236.71±108.29	MC = 701.01±38.21<br>SC = 358.06±116.29
Length of glandular trichomes (µm)	GI = 229.57±87.41	GI = 275.99±107.84	-	GII = 98.50±85.67
**LEAVES ARCHITECTURE** (Fig. 7)				
Major secondary vein framework	Festooned brochidodromous	Festooned brochidodromous	Festooned brochidodromous	Festooned brochidodromous
Variation of secondary angle	Smoothly decreasing proximally	Smoothly decreasing proximally	Smoothly decreasing proximally	Smoothly decreasing proximally
Major secondary attachment	Excurrent, basally decurrent	Excurrent, basally decurrent	Excurrent	Excurrent, basally decurrent
Intersecondary proximal course	Parallel, sometimes perpendicular	Parallel, sometimes perpendicular	Parallel	Parallel
Intersecondary distal course	Reticulate	Reticulate	Reticulate	Reticulate
Intersecondary vein frequency	<1 per intercostal area	<1 per intercostal area	<1 per intercostal area	<1 per intercostal area
Exmedial course	Parallel to intercostal tertiary	Parallel to intercostal tertiary	Absent	Absent
Exterior course	Basiflexed	Basiflexed	Absent	Absent
Exterior tertiary course	Looped	Looped	Looped	Looped
Quaternary and quinternary vein fabric	Irregular reticulate	Irregular reticulate	Irregular reticulate	Irregular reticulate
