# Supplementary material for: Integration of leaf traits supports the current circumscription of Afgekia Craib. and Padbruggea Miq. (Fabaceae, Wisterieae)
Source: PhytoKeys. 2026 Feb 5;270:289–323. doi: 10.3897/phytokeys.270.181424 (PMC12902768; doi:10.3897/phytokeys.270.181424)
Supplement: Supplementary material 2 — Phylogenetic placement of Afgekia (pink) and Padbruggea (blue) within the tribe Wisterieae, based on Compton et al. (2019) [file phytokeys-270-289_article-181424__-s002.pdf]

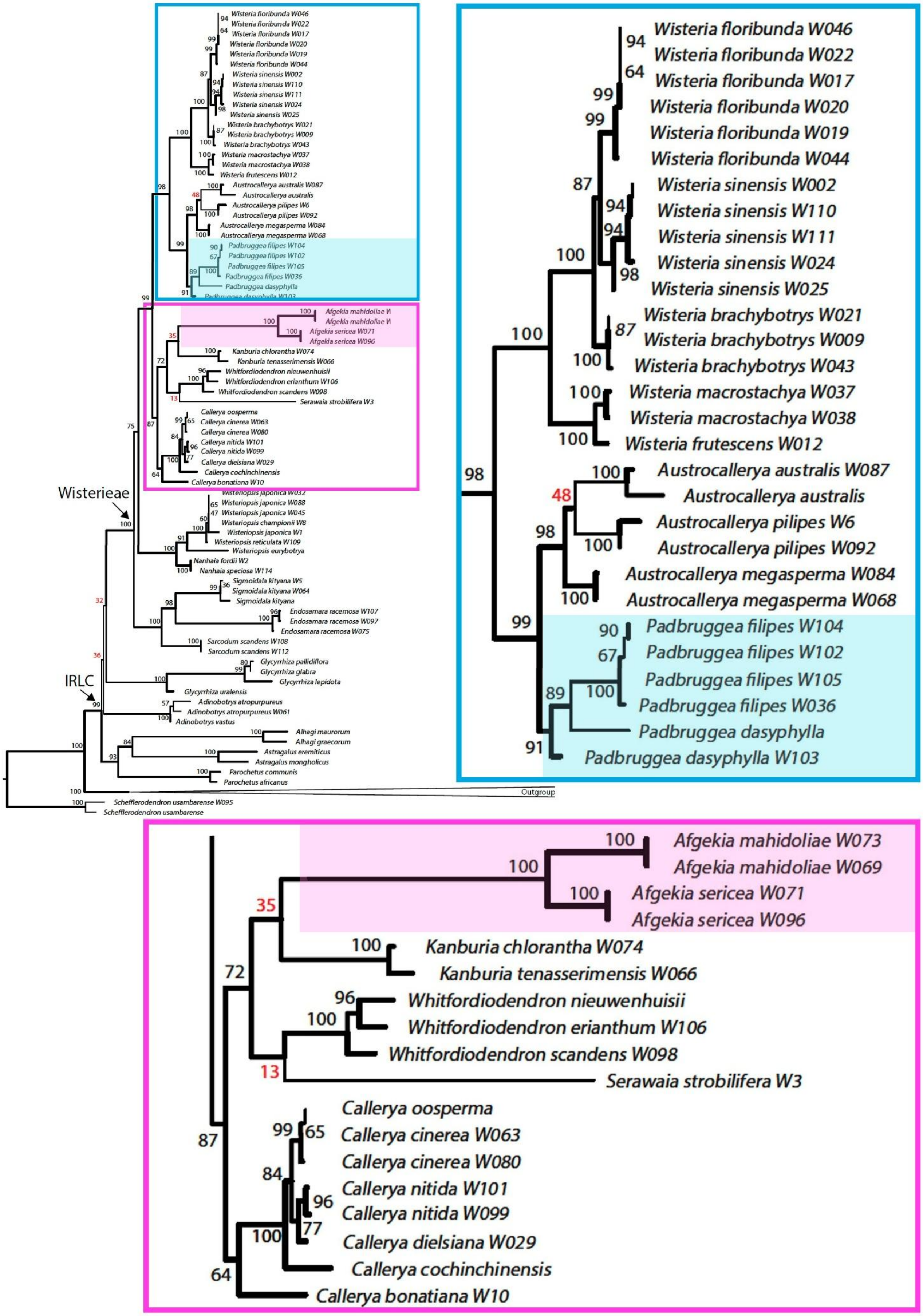

Figure S1. Phylogenetic placement of *Afgekia* (pink) and *Padbruggea* (blue) within the tribe Wisterieae, based on Compton et al. (2019).
